# Reply to Mr. Oliphant's Information on Mrs. Craib's Case of Cancer

**Published:** 1801-01

**Authors:** William Nisbet

**Affiliations:** Fellow of the Royal College of Surgeons, Edinburgh, &c.


### *Reply to Mr. Oliphant's Information on Mrs. Craib's Case of Cancer*; *by* William Nisbet, M.D. Fellow of the Royal College of Surgeons, Edinburgh, &c.

To the Editors of the Medical and Physical Journal.

Gentlemen,

IN the Nineteenth Number of your Journal, was inferted a cafe of the cure of Mammary Cancer, as drawn up by Dr. Nifbet. The relation of that cafe has excited much attention, and much medical fcepticifm has been betrayed on the fubject; to favour which, in your laft number, a new ftatement has even appeared, under the title of *information* refpecting the cafe of Mrs. Craib, by Mr. Oliphant the attending furgeon. As the title of this ftatement is clearly meant to imply a reflection on the former relation, it is my duty to reply to it fully; and to fhow, that the facts *then* advanced, and the principles of treatment then detailed, were equally juft, and deferving the notice of the public.

In making this reply, I fhall firft examine the defcription of the difeafe prefented by Mr. Oliphant; 2dly, the principles affumed by him as effecting the favourable termination of it; and, 3dly, remark on the analogous cafes cited by him in fupport of thefe principles.

With refpect to the defcription of the difeafe, little difference occurs to be noticed in the two ftatements; indeed, the morbid appearances were fo ftrongly marked and characteriftic, as not to admit of any. At the fame time it will be apparent to every unprejudiced reader, how much, even in this part, Mr. Oliphant has endeavoured to ftrain every fact to favour his particular opinion: Every real cancer he confiders as incurable, and no difeafe he regards as cancer that is curable. Laying down then this axiom, the malignity of the morbid appearances in the prefent inftance, which could not admit denial, he afcribes lefs to the fpecific nature of the malady than to preceding errors in its treatment. But in this attempt, like moft people who deviate from the fair or candid line of conduct, he has placed himfelf in a moft aukward dilemma, and by fetting up his fingle ipfe dixit, has oppofed the opinions of fome of the moft refpectable of the profeffion. He has found it neceffary, therefore, to omit altogether the names of the gentlemen formerly concerned with the cafe, and whofe fentiments he was made acquainted with by the patient, as well as Dr. Nifbet, fufficing

ſufficing himſelf with giving Mr. Cline the appellation of a competent ſurgeon, and Sir James Earle of an eminent and experienced one, but taking no farther notice of any other perſon. Whether theſe gentlemen will be more offended by their names being formerly noticed as having merely ſeen the caſe, or now being declared ignorant of its real nature, and as having paſſed an erroneous judgment upon it, it is not my buſineſs to determine: their names became introduced by me in conſequence of the patient's own relation, and *that* with propriety, in order that they might have an opportunity of proving the authenticity of my ſtatement. I conſider them alſo, men of too much liberality and candour, and too much actuated by zeal for the improvement of ſcience, to ſuppoſe them capable of taking offence on ſuch grounds. The juſtice of their opinion I never could have a doubt of; for if ever there was a caſe of real idiopathic cancer, it was clearly exemplified in this inſtance: The diſeaſe had made its appearance at the cancerous, or critical period of life; it had occurred in a conſtitution ſubject to rheumatic complaints, the connection between which and future cancer is ſo well pointed out by the late Dr. Fothergill; and it had attacked more than one part of the ſyſtem, without any injury being urged as giving origin to it in this ſecond inſtance; it poſſeſſed alſo, every pathognomic appearance of cancer to the examination of the ſurgeon, and every feeling of it to the patient herſelf; nor to this hour would any doubt have even ariſen in Mr. Oliphant's mind reſpecting its nature, had it remained incurable. How trifling muſt his remark appear, which aſcribes its malignity to a cataplaſm of pigeon's dung, applied by the patient's friend! Such an application could have no bad effect, without a primary inherent cancerous tendency in itſelf.

From theſe remarks on the deſcription of the diſeaſe, I ſhall now examine the principle of cure, and on this principle no difference of opinion can exiſt. That a conſtitutional diſeaſe will at one time ſuſpend, and at another time entirely cure a local affection, is a fact which every tyro in medicine is acquainted with; but though acquainted with it as an acknowledged fact, much obſervation and experience are requiſite where this exertion of the powers of Nature is wiſhed to be imitated with ſucceſs. Indeed, the proper application of this circumſtance, leads to a ſucceſsful cure of moſt chronic diſeaſes; but in order to this ſucceſs, the means employed will require to be as varied as the nature of the diſeaſe, or chronic affection, which is to undergo the change. It is on this principle entirely, I conceive the cure of lues, or the conſtitutional venereal venereal difeafe to proceed: the action of mercury is properly nothing more than changing it from a chronic to an acute form, fimilar to fmall-pox and meafles, or exciting and continuing a phlogiftic action or pyrexia, till the expulfion of the difeafe. This opinion I fuggefted fifteen years ago, in a treatife on that fubject; fince that time I have found no reafon to change my fentiments, in fpite of the numerous chemical hypothefes and evanefcent theories of the day. The operation of remedies, I am clear, can only be juftly explained on principles confonant to the laws of the animal œconomy; and the fubjecting an animated machine to the laws of inanimate matter, is lofing fight of the power of vitality, and the peculiar energies of life, its leading diftinctions.

Agreeing then fully with Mr. Oliphant in the general principle laid down, that the conftitutional difeafe, in the prefent cafe, was the means of curing the local one, the only queftion betwixt us is, Whether this conftitutional difeafe was the effect of accident or art? In order to make it the former, the fame difingenuous attempt is fhown on Mr. Oliphant's part, as in the defcription of the morbid appearances; to detect which, it is only neceffary for me to detail the particular courfe I employed.

When I was firft called in to fee Mrs. Craib, the cafe appeared, as Mr. Oliphant juftly obferves, a hopelefs one, and palliation was all the relief to be expected: The opinion I then gave, will mark, at leaft, the formidable nature of the malady, and prove, that Mr. Oliphant had no *favourable* doubts at that period. I prefcribed for her a diet drink and pill; the former, with a view to produce fome change in the ftate of the fluids; the latter, for the particular purpofe of exciting the action of the lymphatic fyftem: an opiate was alfo enjoined to be occafionally ufed, as indicated by the urgency of pain. To the fore itfelf, a dreffing of common cerate was applied, and the fermenting poultice laid afide at my defire, from the pain and irritation it conftantly produces in every cafe of cancer. In this courfe the patient proceeded. Every day, according to her own account, fhe felt greater eafe and abatement of pain, till the latter entirely ceafed; and, as fhe expreffed herfelf, fhe would not have known from her feelings any difference betwixt the ulcerated and the other breaft. She could now lie with eafe on the affected fide, and ufe that arm with freedom; circumftances to which, previous to the commencement of this courfe, fhe was a ftranger. The breaft itfelf fhowed an evident fhrinking of fize; and Mr. Oliphant at that period obferved, it was clear the progrefs of the difeafe was now *completely* checked. Reflecting on this favourable appearance, and defirous ous that it might proceed more ſpeedily, I now ſuggeſted to Mr. Oliphant the propriety of employing frictions, ſo as to ſend the ſame remedies directly through the diſeaſed part, without the circuitous courſe of the ſyſtem at large. To this meaſure he objected, from the ſtate of the weather and their ſuppoſed inefficacy; confident, however, in my own opinion, they were applied according to my direction by the perſon mentioned in his ſtatement, as having formerly attended the patient as a friend. This practice was continued for the ſpace of ſix weeks, in the courſe of which time, not only was the regular ſhrinking of the breaſt conſpicuous both to that perſon and the patient, but ſeveral of the ſmaller tumours ſurrounding the diſeaſed maſs were entirely abſorbed; facts, for the truth of which I appeal to themſelves.—So completely ſatisfied was the patient of this, that not content with the friction at night, as employed by her friend, ſhe had recourſe to it herſelf in the day, and thus it was carried beyond what was neceſſary for the actual cure of the diſeaſe. The ſubſequent pleuritic attack, therefore, may be conſidered in part, perhaps, as the conſequence of her own imprudence, and it was attended with this farther miſchief of interrupting the courſe too ſoon. During this time the ſchirrous breaſt ſhrunk in proportion to the other, but never ſhowed thoſe ſtrong marks of yielding ſoftneſs till the courſe was at its heighth, at which period, even external ſymptoms of inflammation were conſpicuous on its ſurface; and when examined ſome time after by the gentlemen who ſaw her, it was ſo ſoft as hardly to bear the name of ſchirrous, and in a daily decreaſing ſtate. From this period, the management of the caſe, at Mr. Oliphant's ſpecial deſire, was left entirely to himſelf; nor was I either conſulted, nor, except from hearſay, knew what was employed. What I ſee of the practice, now ſufficiently ſhows me the impropriety of the applications; but as it is his wiſh to claim all the merit of the caſe to himſelf, and for that purpoſe his ſtatement was written, it is not my buſineſs to interfere with the ſubſequent treatment. From the detail of the prior courſe, it will be ſeen, that the progreſs of cure in this diſeaſe was gradual, regular, and bore a proportion to the activity of the means employed; that the relief experienced was from the commencement of this courſe, and that the conſtitutional diſeaſe was the conſequence of the means employed, although aggravated by the imprudence of the patient herſelf, in a degree beyond what was neceſſary, and perhaps, alſo, by the accidental cauſes which Mr. Oliphant takes notice of: The termination, however, has been favourable, and would have been much more complete, but for the intervention of theſe cauſes. On the whole, neither the patient tient herfelf, nor any perfon who faw her in her worft, and apparently incurable ftate, will agree with the propriety of Mr. Oliphant's quotation from Amatus Lufitanus, that it is better to let the difeafe alone. In making this quotation, he might have gone even a good deal higher, for he will find the fame obfervation a trite adage from the time of Hippocrates downwards, though, if it has any truth in it, it applies more properly to the early than advanced ftage of the affection.

After this view of the cafe, it remains that I fhould examine the analogous cafes, cited by Mr. Oliphant, in fupport of his ftatement. All his cafes are evidently of a fcrophulous nature; they were cured, the worft of them, by bark and carbonic acid, medicines which will always, in real cancer, do harm. The only one that applies in any degree to that of Mrs. Craib, from its occupying the fame fituation, is the cafe of Mrs. Ofborn; but here the procefs of cure, when examined, is materially different.

In Mrs. Ofborn's cafe, Nature, as it were, fhrinking from itfelf, and unable to fupport a load, allows it firft to lofe its vitality, and then to feparate; and on this feparation fuch predominant weaknefs prevails, that the veffels are even unable to contract, or prevent, exceffive hæmorrhages, which continue for a length of time. The fkin alfo, in healing, becomes puckered in the fame manner as in the healing of other fcrophulous ulcers. Nor, during this procefs, is the axiliary fwelling removed, but it continues for a vaft length of time in a difeafed ftate. Where, then, is the comparifon between this and Mrs. Craib's cafe? In the latter, a general increafed action of the fyftem appeared; and from the moment of its appearance, the procefs of abforption was ftrong and complete over the difeafe. No floughing or feparation took place, for the action of the abforbents was equal to the removal; and the time in which this procefs was accomplifhed, difplayed the prefence of an active and daily increafing caufe, goading on Nature in her efforts, and threatening even injury to the fyftem, in order to its fuccefs.

If inanition formed the principle of cure in fcrophula and cancer, as contended for by Mr. Oliphant, it is evident thefe difeafes, among the lower claffes at leaft, fhould be very readily cured; for Nature could never be at a lofs to have abundant opportunities of employing her exertions on the enfeebled, worn-out, conftitutions of fuch patients. But is there an inftance of this fort to be met with? Do not thefe difeafes make much more rapid progrefs amongft the lower claffes? And are not, alfo, the lower claffes the moft frequently attacked by them?"

Before

Before concluding, I ſhall only beg leave to mention, that when the ſtatement of Mrs. Craib's caſe was to be made out, I mentioned the propriety of it to Mr. Oliphant, in which he readily acquieſced. When it was afterwards made out, it was read to him by me, and he ſuggeſted one alteration, which, at his deſire, I adopted; viz. that the cure was effected "without ſloughing by an increaſed abſorption, in which it differed from any he had ever ſeen." According to every idea I could then form of a man's ſentiments, the account drawn up by me, with this one alteration, had his unqualified approbation, and I even then told him it ſhould go in our joint names. On this conduct I ſhall make no comment; the matter is before the public. Three more caſes will be publiſhed by me in the courſe of a few weeks. Theſe will ſufficiently determine the point, and be the beſt anſwer to all the ſcepticiſm diſplayed on the ſubject.

In bringing Mrs. Craib's caſe into notice, it was my wiſh to call the attention of practitioners, in a particular manner, to this diſeaſe; and in doing ſo, to ſhow,

1ſt. That the general principle of exciting phlegmonic action to a certain degree, and continuing it, is ſufficient to cure cancer, whether in the ſtate of ſchirrus or ulceration.

2d. That the preferable mode of exciting this action is through the medium of the lymphatic ſyſtem.

3d. That the lymphatic ſyſtem, once excited, is capable of producing abſorption, equal to the removal of the moſt hardened maſs of diſeaſe.

4th. That the cure is to be ſought for in a proper ſelection of thoſe ſubſtances which are moſt powerful in exciting the lymphatic ſyſtem, and which continue this excitement in a permanent manner. I am,

Gentlemen,

Your obedient ſervant,

W. NISBETT.

*St. James's Street,*

